# Assessing the efficiency of health systems in achieving the universal health coverage goal: evidence from Sub-Saharan Africa

**DOI:** 10.1186/s13561-023-00433-y

**Published:** 2023-05-02

**Authors:** Kwadwo Arhin, Eric Fosu Oteng-Abayie, Jacob Novignon

**Affiliations:** 1 Department of Economics, Ghana Institute of Management and Public Administration, Accra, Ghana; 2 Department of Economics, Kwame Nkrumah University of Science and Technology, Kumasi, Ghana

**Keywords:** Universal health coverage, Health system efficiency, Health service coverage, Financial risk protection, Sub-Saharan Africa

## Abstract

**Objective:**

Universal health coverage (UHC) is a major pathway to save many people from catastrophic and impoverishing healthcare spending and address the inequality in health and healthcare. The objective of this paper is to assess the efficiency with which health systems in sub-Saharan Africa (SSA) are utilizing healthcare resources to progress towards achieving the UHC goal by 2030.

**Methods:**

The study followed the guidelines proposed by the World Health Organization (WHO) and World Bank joint UHC monitoring framework and the computational operationalization approach proposed by Wagstaff et al. (2015) to estimate the UHC index for each of the 30 selected SSA countries. The bootstrapping output-oriented data envelopment analysis (DEA) was used to estimate the bias-corrected technical efficiency scores and examine the environmental factors that influence health system efficiency.

**Results:**

The estimated UHC levels ranged from a minimum of 52% to a maximum of 81% $(SD=8.6\%)$ with a median coverage of 66%. The average bias-corrected efficiency score was 0.81 $\left(95\% CI:0.77-0.85\right)$. The study found that education, governance quality, public health spending, external health funding, and prepayment arrangements that pool funds for health had a positive significant effect on health system efficiency in improving UHC, while out-of-pocket payment had a negative impact.

**Conclusion:**

The results show that health systems in SSA can potentially enhance UHC levels by at least 19% with existing healthcare resources if best practices are adopted. Policymakers should aim at improving education, good governance, and healthcare financing architecture to reduce out-of-pocket payments and over-reliance on donor funding for healthcare to achieve UHC.

**Supplementary Information:**

The online version contains supplementary material available at 10.1186/s13561-023-00433-y.

## Introduction

The 2005 World Health Assembly Resolution urged national governments to work towards universal health coverage (UHC) so that all people, including the poor, have access to essential health services and do not suffer financial hardship paying for them [1]. Since this pledge was made, national governments and development partners have embraced the principle of UHC and are pursuing varying programs and policies to reduce physical and financial barriers that exclude the poor and marginalized from accessing essential healthcare services [2–5].

The adoption of the Sustainable Development Goal 3 (SDG 3) in the year 2015 has marked a shift of focus in the global and regional discourse on health from the elimination or reduction of specific health conditions to universal health coverage (UHC), which is the Sustainable Development Goal (SDG) target 3.8 [6]. Achieving UHC is recognized as both an end in itself as well as the major means to attaining other health-related and non-health-related SDG targets. World Health Organization (2010) estimates that about 150 million around the globe face catastrophic health expenditures due to high out-of-pocket health expenditures [7]. UHC is a major pathway to saving the poor from catastrophic health expenditure, which is estimated to plunge approximately 100 million people into poverty every year [8].

Most SSA countries have integrated UHC as a goal in their national health strategies. Leaders of SSA have demonstrated their continued commitment in support of stronger health systems that speed up the progress towards the attainment of the UHC goal as evidenced in the declarations in Abuja (2001), Ouagadougou (2009), and Luanda (2014). The average level of per capita public spending on health has more than doubled from about US$70 in the early 2000s to more than US$160 in 2014 (in purchasing power parity terms), albeit below the Abuja Declaration target of allocating 15% of the annual budget to health [9]. Many countries in the region are currently engaged in health reforms that aim at extending and improving coverage of essential health services and/or financial protection [10–12]. The UHC agenda offers a system-wide implication across the full spectrum of health services, presenting a unique opportunity to SSA countries to drive progress toward better health results in the region.

In the years before the SDGs, the SSA region made big strides during the MDG era: preventable child deaths plummeted by more than half, and maternal mortality went down by almost as much [13]. Despite this modest achievement, a World Bank report [14] reveals that some numbers remain high, like the fact that five million children still die every year before their fifth birthday, or that HIV-AIDS is the leading cause of death for adolescents in the SSA. Many countries in SSA still contend with high levels of maternal deaths, malnutrition, and a growing burden of chronic diseases such as diabetes, cancer, and stroke [15]. The whole region is simultaneously experiencing multiple epidemics – Ebola, COVID-19, HIV, tuberculosis (TB), and non-communicable diseases (NCDs).

These challenges call for accelerated progress towards the UHC goal to ensure that everyone receives all the necessary health services without suffering financial hardship. The World Health Report [16] identified inefficient use of resources as one of the three major factors that impede progress toward the UHC goal. The report conservatively estimated that between 20 to 40 percent of resources spent on health go waste due to inefficiency. Efficiency measurement of health systems is important to assess the extent to which scarce healthcare resources are used to get the best value for money and serves as a major criterion for priority setting [17, 18]. It is one of the three facets of assessing the overall performance of health services [1, 19].

However, the efficiency of health systems in achieving the UHC goal has received little attention in the literature [20]. Previous studies have focused on the spending efficiency of specific diseases [21–26] and the efficiency of health systems in general [27–30]. From the accessible literature and to the best of our knowledge, only one cross-country study has estimated UHC and evaluated the efficiency of health systems in achieving the UHC goal [31]. This analysis was for 172 countries using 2015 data.

This current study extends the frontiers of the previous studies and differs significantly in three major ways. First, this study strictly adopts the joint WHO and World Bank framework on measuring UHC [6]. Second, the study adjusts the health service coverage indicators for inequality in access to healthcare services. Third, this study uses the complements of catastrophic and impoverishing health expenditure as the financial protection indicators in measuring UHC. The purpose of this study is in three-fold: (i) to estimate the level of UHC achieved by health systems in sub-Saharan Africa using the joint WHO and World Bank framework, (ii) to measure the technical efficiency of the health systems in achieving the UHC goal, and (ii) to examine the factors that explain differences in the efficiency levels.

The rest of the paper proceeds as follows: Methods and Materials section presents the methods and data used in the analyses of the study. Empirical Results and Discussions section discusses the results while Conclusion and Policy Implications section concludes the study with some policy implications.

## Methods and materials

### Material and data

This study adopted the conceptual framework proposed by the World Health Organization [1] which links health inputs with health outcomes (or outputs). The health system framework identifies six major building blocks that must be laid at the foundation of every well-functioning health system to achieve four overall outcomes (or goals). The six system building blocks identified in the framework are health service provision, health workforce, information systems, medicines and technologies, health financing, and governance and leadership. The framework defined the four overall outcomes or goals as improved health (level and equity), responsiveness, financial risk protection, and improved efficiency.

This study focuses on health workforce and health expenditure as the key health system inputs underlying the health production function that are used to estimate health system efficiency levels [32, 33]. Meanwhile, health service coverage indicators (in both level and equity) and financial risk protection coverage indicators are used as the intermediate health system outputs [34]. The study assumes that the level of UHC attained by a country depends on the amount of healthcare resources dedicated to that purpose.

In a typical health systems efficiency study, health outcomes such as life expectancy at birth, health-adjusted life expectancy (HALE), disability-adjusted life expectancy (DALE), and mortality rates are used as proxies for output variables to represent the general health of the population [35–39]. However, in the current SDGs-era health systems deploy healthcare resources with the major aim of achieving UHC targets and indicators, particularly in the study setting of the SSA region. Again, UHC indicators perform better in reflecting the general health of the population than health outcomes such as health-adjusted life expectancy (HALE), disability-adjusted life expectancy (DALE), and mortality rates. Thus, this study used UHC indicators as the output variables since they have a more direct and relevant relationship with national expenditure on health. Many countries accept UHC as a principal goal for their health care systems and it presents an exceptional prospect to promote an all-inclusive approach to health, beyond the treatment of specific diseases, to focus on the full spectrum of health services to drive progress towards better health outcomes in the SSA region [9, 40].

UHC, as defined by WHO/World Bank, has two main dimensions: service coverage (all people – irrespective of their socioeconomic background – are getting the services they need) and financial risk protection (no one suffers financial hardship because of seeking needed health care) [6]. Drawing motivation from the United Nations Development Programme (UNDP) Human Development Index, many researchers make use of indices to summarize information about an underlying health construct. In the literature, the use of indices to represent the output of a health system is becoming increasingly popular [41–43]. In line with the main objective of this study, we employed the two main components of UHC, the service coverage (SC) index and the financial risk protection (FP) index, to construct the UHC index as the single output variable. We followed the guidelines proposed by the WHO and World Bank joint UHC monitoring framework [6] and the computational operationalization approach proposed by Wagstaff et al. [42].

### Service coverage index

In this study, ten core tracer health service coverage (SC) indicators were used for reproductive, maternal, newborn, and child health (family planning, antenatal care, skilled birth attendance, and full immunization); infectious disease (HIV antiretroviral treatment, tuberculosis treatment, diarrhea treatment, and insecticide-treated bed-net); and non-communicable disease (acute respiratory infection treatment). We selected these coverage indicators because they represent interventions from which every individual in every country of the SSA region should benefit regardless of that country’s level of socio-economic development and epidemiological circumstances. Again, the study chose these indicators because there were comparable data for most countries. Thus, the selected ten tracer service coverage indicators met the criteria related to relevance, feasibility, effective coverage, and usability set forth by the joint WHO/World Bank framework for monitoring intervention coverage [6, 42, 44].

Following the UHC monitoring framework, we grouped the spectrum of the ten chosen health service interventions into two broad categories: *prevention* (which includes health promotion and illness prevention) and *treatment* (which includes curative treatment, rehabilitation, and palliation). For prevention services coverage $({SC}_{P})$, we employed five tracer indicators: antenatal care visits of at least four visits (ANC), full immunization (IMM), insecticide-treated bed-net (ITN), contraceptive prevalent rate (CPR), and satisfaction of family planning needs (FAP). We selected another five tracer interventions for treatment services coverage $({SC}_{T})$: tuberculosis treatment (TB), HIV antiretroviral treatment (ART), diarrhea treatment (DIA), acute respiratory infection treatment (ARI), and skilled birth attendance (SBA). We excluded other SC indicators recommended in the joint World Health Organization (WHO) and World Bank (WB) framework because data were not available to allow comparisons across countries. The idea of the SC index is to count the number of people in *need* (denominator) out of which a given number of people are receiving (numerator) the health service interventions [42].

In addition to capturing *need*, we adjusted the service coverage indicators for *inequality* between the poor and the better off by converting from the population mean to an ‘achievement’ index [35]. At the heart of UHC is the commitment to ensure equity—that all people who need health services can access them without suffering financial hardship [6]. Thus, we included a dimension of inequality by switching from the population mean to the achievement index to ensure that countries that have disproportionately lower coverage of health service intervention among the poor are ‘penalized’. That is, the achievement index becomes lower than the population mean for a country with disproportionately lower coverage of the health service intervention among the poor relative to the rich, and vice versa. Following Wagstaff et al. [42], the achievement index (*AI*) for each health service coverage indicator is computed as the population mean $(\overline{X })$ multiplied by the complement of the concentration index (*CI*) as shown in Eq. (1).

1 $$AI=\overline{X }(1-CI)$$

We aggregated the five prevention indicators scores into a single summary score using the geometric mean of the prevention indicators scores giving each indicator equal weight as in Eq. (2).

2 $${{SC}_{P}=[ANC.IMM.ITN.CPR.FAP] \, }^{{}^{1}\!\left/ \!{}_{5}\right.}$$

Similarly, we the computed geometric mean of the treatment indicators, weighting each indicator equally, as indicated in Eq. (3).

3 $${{SC}_{T}=[TB.ART.DIA.ARI.SBA] \, }^{{}^{1}\!\left/ \!{}_{5}\right.}$$

We then computed the geometric mean of the prevention services coverage index (${SC}_{P}$) and the treatment services coverage index (${SC}_{T}$) to obtain the overall level of service coverage (*SC*) in line with the approach by Wagstaff et al. [42]. Based on Kaplan et al. [45] who estimated an average of 75% and 25% as the share of treatment domain and prevention domain, respectively, in the total health spending in the SSA, we assigned a lower weight of 0.25 to the prevention indicator and higher weight of 0.75 to the treatment indicator as shown in Eq. (4).

4 $$SC={SC}_{P}^{0.25}.{SC}_{T}^{0.75}$$

### Financial risk protection index

Two indicators that the UHC monitoring framework proposed and are commonly used to track the level of the financial protection in seeking healthcare services are the incidence of catastrophic health expenditure (*CATA*) and the incidence of impoverishment due to out-of-pocket health payment (*IMPOV*) [6, 43]. Out-of-pocket payment for health care is deemed ‘catastrophic’ when it exceeds a certain threshold of household consumption or expenditure, while ‘impoverishment’ captures the extent to which out-of-pocket payment for healthcare services pushes a household below the poverty line [46, 47]. In the literature, the threshold used to define catastrophic payments varies, such as 10%, 15%, 20%, and 25% of total expenditure. Similarly, we have different internationally accepted poverty lines, such as US$1.25, US$1.90, and US$3.20 per day per capita (at purchasing power parity) consumption used by the World Bank. In this study, we chose a catastrophic spending threshold of 10% of total consumption and a poverty line of US$1.90-a-day for the impoverishment indicator.

The two indicators measure the lack of financial protection in seeking health care. The joint World Health Organization (WHO) and World Bank (WB) framework for monitoring progress toward UHC proposed that financial protection (FP) indicators be rescaled so that 100% coverage represents full financial protection [6]. In addition, efficiency assessment considers higher values as desirable. Based on these reasons, we followed the approach by Wagstaff et al. [42] and computed the complements of the two financial protection indicators, i.e., fraction or proportion of households that did not incur catastrophic (*1 – CATA*) and impoverishing (*1 – IMPOV*) health care payment. We measured both indicators on a scale of 0 to 100% with 100% representing full financial risk protection [6]. Again, given that policymakers are concerned with both financial protection indicators, and are presumably willing to trade off one against the other at a diminishing rate, we weighted equally and computed the geometric mean of the two indicators into single financial risk protection (FP) index as in Eq. (5).

5 $$FP={[\left(1-CATA\right)\left(1-IMPOV\right)]}^{{}^{1}\!\left/ \!{}_{2}\right.}$$

We noted that the two FP indicators of incidence of impoverishment and catastrophic expenditure only cover the share of the population who incurred out-of-pocket (OOP) payments for health. The two indicators fail to account for those who were discouraged from seeking health services because the cost of doing so was simply unaffordable, which in the study setting of SSA may account for a significant share of the population. One way to resolve this problem is to establish the *need* for specific health service interventions, which we can do with the health coverage indicators. This underscores the importance of monitoring health service and financial protection coverages concurrently and side-by-side [6].

### UHC index

Following Wagstaff et al. [42] and Barasa et al. [20], we computed the UHC index by aggregating the service coverage index (*SC)* and the financial protection index (*FP)* in Eqs. (4) and (5), respectively, giving each domain equal weight as shown in Eq. (6).

6 $$UHC={[SC.FP]}^{{}^{1}\!\left/ \!{}_{2}\right.}$$

**Fig. 1:**
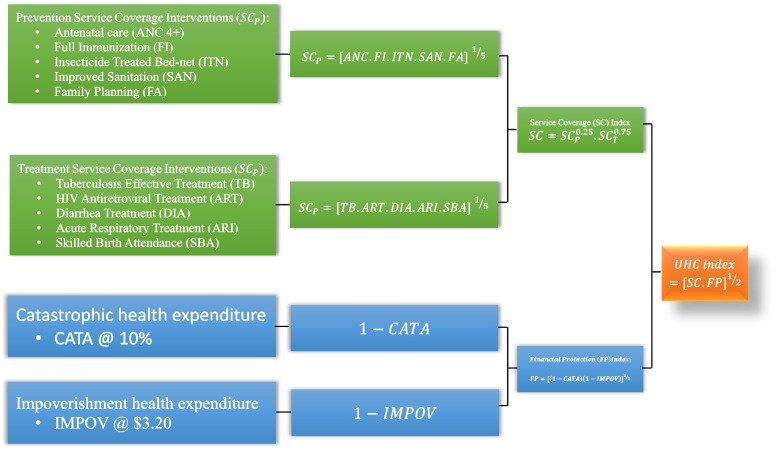
Universal Health Coverage (UHC)

Figure 1 presents the summary of the conceptual framework for the computation of the UHC index. In this study, we utilize the UHC index as the output variable for the health system efficiency analysis, while health expenditure, physician density, nurses and midwives density, and hospital bed density are used as the input variables. Appendix A presents the *numerator* and *denominator* definitions of all the health service coverage (SC) and financial risk protection (FP) indicators used in this study.

### Data source

We sourced the data used for the analysis of this study from the World Bank’s new database called Health Equity and Financial Protection Indicators [48]. The health service interventions and the financial protection coverage indicators in the database are generated from well-known household surveys that have been conducted by, or in partnership with national governments, such as the Demographic and Health Surveys [49], the Living Standards Measurement Surveys [50], and Multiple Indicator Cluster Surveys [51]. We also sourced data from World Development Indicators [52] and World Health Organization’s Global Health Observatory [53].

To take advantage of experts and ensure consistency, we gleaned the estimates for service coverage, concentration indices, and financial protection indicators from World Bank and World Health Organization databases. Health coverage indicators are estimated mainly from Demographic and Health Surveys (DHS) and Multiple Indicator Cluster Surveys (MICS) while financial protection indicators are largely estimated from Living Standards and Measurement Surveys (LSMS) [42]. Since these two main domains of indicators for the computation of UHC typically come from different surveys conducted in different years, obtaining a UHC index for a particular year is usually difficult. We remedied this challenge of data blind spots by computing the UHC index using recent surveys and indicating the median survey year. This study chose 2015 as the median year. If estimates were not available for 2015, estimates for the most recent previous or following year’s estimates were used [42]. Thus, the most recent estimate between 2012 and 2018 was used if no estimate was available for the year 2015.

According to the World Bank list, there are 48 SSA countries and territories. Among these, 30 countries were used for this study due to missing data. Some selected variables for the study were not reported for the median year of 2015. This is a common problem with World Bank’s World Development Indicators (WDI) and Health Equity and Financial Protection Indicators (HEFPI) databases [54–56]. In the literature, there are several approaches for dealing with a missing data problem. One approach is to use a value from slightly earlier or later years as in Anderson et al. [54] and Ahmed et al. [56]. An alternative approach is to exclude countries (or observations) with missing data resulting in a smaller number of countries in the model as in Fare et al. [55]. This study opted for the former approach since the DEA methodology employed in this study requires that there are as many decision making units (countries) as possible (at least three times the number of inputs and outputs) to enhance the discriminating power of the model.

### Data envelopment analysis

Charnes, Cooper, and Rhodes (CCR) [57] originally proposed DEA to estimate the relative efficiency of a set of decision-making units (DMUs) that use comparable inputs to produce a set of outputs. In estimating the efficiency frontier, Charnes, Cooper, and Rhodes (CCR) assumed constant return to scale (CRS) production, which means a proportionate increase in the level of inputs, causes the same proportionate increase in the level of outputs. The constant return to scale (CRS) assumption is appropriate only when all the decision-making units (DMUs) are operating at their optimal scale. Thus, the constant return to scale (CRS) assumption may not be practical when DMUs operate at a suboptimal scale due to imperfect competition, government regulations, public sector planning bureaucracy, and constraints on a budget.

Banker, Charnes, and Cooper (BCC) [58] extended the CCR model to account for variable returns to scale (VRS). The VRS assumption implies that any increase in the level of inputs would proportionately increase or decrease the level of output. This study adopted the Banker, Charnes, and Cooper (BCC) DEA model as it is assumed to be more appropriate in healthcare systems efficiency studies since the health services production process is considered to be an increasing concave function of health expenditure.

Further, DEA models in healthcare systems efficiency studies are specified as either input-oriented or output-oriented [37, 56]. An output-oriented DEA model seeks to maximize the outputs with a given set of inputs, while the input-oriented DEA model aims to minimize inputs for a constant amount of outputs. This study adopts the output-oriented DEA methodology to assess the efficiency of health systems since in the context of health systems, the output-oriented DEA approach is more appropriate, as healthcare inputs at the national level are unlikely to be changed as health system stewards have little control over budget allocations [34, 59]. Again, in an output-oriented DEA model, technical efficiency assumes that more output, such as higher levels of UHC, is better. Besides, in Sub-Saharan Africa (SSA) region, which serves as the setting for this study, the need for healthcare services are scarcely met. Thus, it would be unethical to reduce the number of healthcare services provided to improve health system efficiency [60]. Besides, the healthcare systems in the region cannot easily reallocate inputs due to large private sector participation and heavy reliance on donor support. According to Oikonomou et al. [61], Cheng et al. [62], and Hernandez and San Sebastian [63], the choice of the output-oriented model in healthcare efficiency analysis is warranted because demand for healthcare services has a leaning to increase and not to decrease. Thus, reducing inputs is objectionable whilst increasing outputs is desirable.

We estimate the efficiency of all the health systems using the VRS output-oriented DEA approach in the envelopment form as given in Eq. (7).

7 $$\begin{array}{c}Max_{\theta,\lambda}\;\theta:\left(x^j,\;\theta y^j\right)\in T\\Subject\;to:\sum\limits_{j=1}^n\lambda_jY_{ij}\geq\theta y_{io},\;i=1,2,\dots,M\\{\textstyle\sum_{j=1}^n}\lambda_iX_{rj}\leq x_{ro},\;r=1,2,\dots,N\\\sum\limits_{j=1}^n\lambda_j=1,\;j=1,2,\dots,K\\\lambda_j\geq0\end{array}$$

where ${DMU}_{o}$ represents one of the K countries’ health systems under evaluation. ${y}_{io}$ and ${x}_{io}$ are the ${i}^{th}$ output and the ${r}^{th}$ input of the ${DMU}_{o}$. $\theta -1$ measures the proportional output expansion that can be attained by the ${DMU}_{o}$, given the input level. Alternatively, $\theta$ measures the technical efficiency (TE) of the health system of the country ${DMU}_{o}$ relative to the technology $T$. The value of $\theta$ ranges from one to infinity. λ represents the weights given to the K health systems which helps determine the envelope formed by the efficient health systems with the restriction $\sum_{j=1}^{n}{\lambda }_{j}=1$ corresponding to the VRS model Banker et al. [58].

### Bootstrap DEA model: stage one

The basic DEA model suffers from statistical limitations, as it does not allow for random error. Thus, the mathematical formulation in Eq. (2) is deterministic since it attributes the distance of any observation to the frontier to only inefficiency. However, in reality, the distance to the frontier reflects both inefficiency and statistical noise as the input–output levels could suffer from measurement error or omission of some of the input–output variables. Recent developments in DEA are capable of estimating the statistical properties of the estimated DEA scores, and thus make it possible to generate an empirical distribution for them. In the first stage, this study adopts the bootstrapping procedure proposed by Simar and Wilson [64] to correct the DEA estimator for bias and to obtain other statistical properties.

### Potential health system efficiency determinants: stage two

In generating the efficiency scores in stage one above, the DEA model captured only discretionary variables that are under the control of the health systems. However, many other characteristics influence the health systems’ output (in this case the level of UHC) but are not captured in the initial stage one DEA analysis. These factors may be endogenous and/or exogenous. Such factors may include governance quality, healthcare services payment arrangements, the level of private sector participation in the provision of healthcare services, government regulatory policies, as well as different macroeconomic and demographic factors. To generate useful insights for policymaking, the study examined the potential associations between the DEA efficiency scores and these health systems characteristics using Kruskal and Wallis [65, 66] test analyses and regressions (including the more usual Tobit approach and the bootstrap procedures suggested by Simar and Wilson [67]).

Following Joumard et al. [68], we classified health systems in SSA into three categories based on the dominant mode of financing healthcare (i.e. state, private, and external); three categories based on the level of economic development (i.e. low-, lower-middle-, and upper-middle-income) countries; and four categories based on geographical location (i.e. eastern, middle, western, and southern) SSA countries. Since DEA is a non-parametric methodology to estimate relative efficiency scores, we could not apply the ANOVA test because DEA does not comply with a normal distribution [36]. Therefore, we conducted the non-parametric Kruskal–Wallis test to verify if statistically significant differences in efficiency existed among these independent groups of health systems.

Conventionally, because the DEA efficiency scores are censored with an upper limit of one, researchers use a simple censored (Tobit) regression to statistically explain the DEA efficiency scores and the potential determinants [36]. However, this approach may cause incorrect statistical inference due to the high correlation between the potential determinants and the discretionary variables (i.e. the output and input variables) used to generate the DEA efficiency scores. This may lead to overstating the precision of the estimates of the potential efficiency determinants, leading to erroneous rejection of the null hypothesis of no statistical association between potential determinants and the DEA efficiency scores. To deal with these econometric issues and ensure consistent and reliable estimates, we employed the bootstraps method proposed by Simar and Wilson [67].

Two major econometric problems we are likely to encounter in carrying out the regression estimations, which may significantly lead to spurious results. First, some of the regressors in the model may be causally related to each other. Thus, separately modeling a regressor without controlling for the effects of the other related regressors on it may cause omitted variable bias. In this study, we dealt with this potential problem by the use of four models to which the regressors suspected to be causally related are added one after another. The full empirical functional model is given in Eq. (8).

8 $${\theta }_{i}={\beta }_{0}+{\beta }_{1}{lnGDP}_{i}+{\beta }_{2}{EDU}_{i}+{\beta }_{4}{AGQ}_{i}+{\beta }_{5}{OOP}_{i}+{\beta }_{6}{GHE}_{i}+{\beta }_{7}{EXT}_{i}+{\beta }_{8}{CFA}_{i}+{\varepsilon }_{i}$$

where ${\theta }_{i}$ is the efficiency score for the ${i}^{th}$ country from solving Eq. (7), $lnGDP$ is the log GDP per capita, $EDU$ is the educational level, $AGQ$ represents the average governance quality, $OOP$ is the out-of-pocket expenditure as a percentage of total health expenditure, $GHE$ represents domestic general government health expenditure as a percentage of general government expenditure, *EXT* represents external sources of financing healthcare a percentage of total health expenditure, and CFA denotes compulsory financing arrangement as a percentage of total health expenditure.

## Empirical results and discussions

### Descriptive summary statistics

**Fig. 2:**
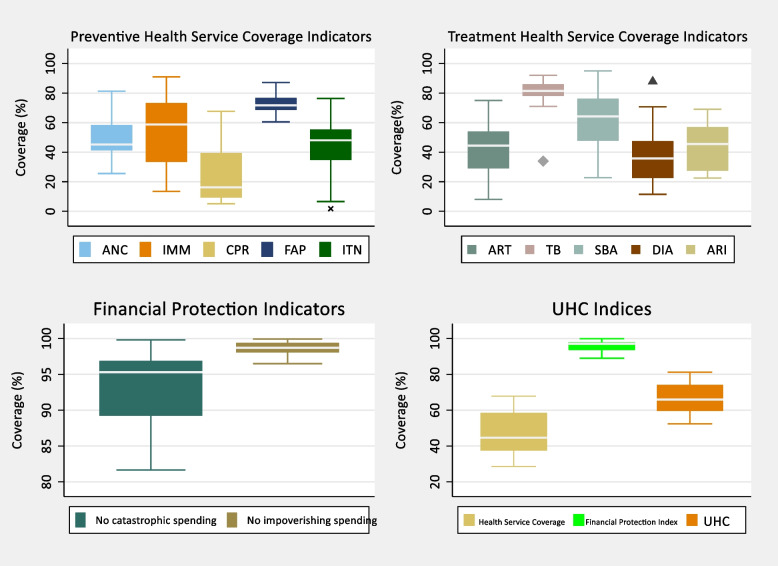
Boxplots of health service and financial protection indicators

Figure 2 shows the distributions of all the indicators. The right and the left upper panels of the figure show the distributions of the five prevention and the five treatment coverage indicators, respectively, used in the study. The figure shows high variation in insecticide-treated bed-net (ITN), full immunization (IMM), contraceptive prevalence rate (CPR), and diarrhea treatment (DIA), with all four indicators having a range from less than 20% to over 60%. On the contrary, family planning (FAP) and tuberculosis treatment (TB) had much lower variation and both had median coverages above 70%. The medians of the maternal and child health (MCH) indicators (antenatal care, full immunization, family planning, and skilled birth attendance) are well below the 80% coverage recommended by Boerma et al. [6]. Overall, only one health service indicator (tuberculosis treatment) achieved a median coverage of more than 80%.

The distributions of the two financial protection indicators presented in the right lower panel of Fig. 2 have a minimum coverage of more than 80%, with the no incidence of catastrophic health spending exhibiting greater variability relative to the no incidence of impoverishment health spending indicator. The median coverage of both indicators is above 95%. The coverage levels of health service and financial risk protection indicators culminated into UHC levels that range from a minimum of 52% to a maximum of 81%, with a median coverage and standard deviation value of 66% and 8.6%, respectively.

**Fig. 3:**
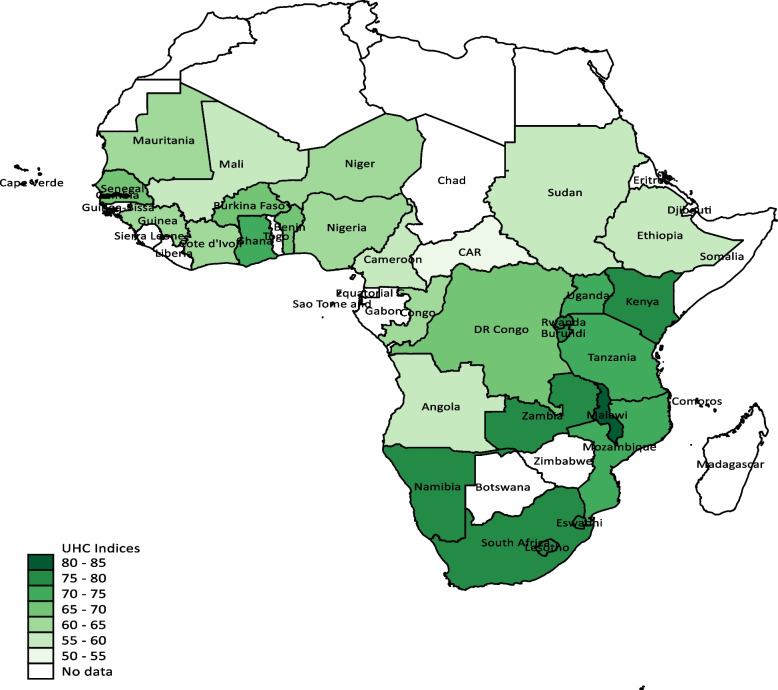
Choropleth map showing spatial incidence of UHC in SSA

**Fig. 4:**
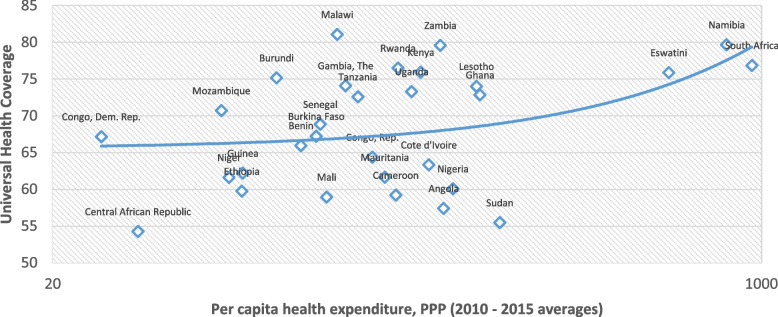
Universal Health Coverage (UHC) and total health expenditure per capita in SSA

The best performers in making progress toward achieving the UHC goal were found in Malawi, Namibia, Zambia, South Africa, and Rwanda (see Fig. 3). These countries offer useful information for the least performing health systems as they are considered good references. They were followed by countries such as Kenya, Eswatini, Burundi, Gambia, Ghana, and Lesotho. At the other end of the spectrum, the Central African Republic, Sudan, Angola, Mali, and Cameroon are the worst-performing health systems when it comes to making progress toward attaining the UHC goal by the year 2030 (see Appendix B for the detailed data on the UHC). Figure 4 shows a scatter plot relating total health expenditure per capita to UHC. A quick inspection of the plot suggests a positive correlation between per capita health spending and UHC.

Table 1 presents the descriptive statistics for the input, output, and environmental variables used in this study. The PPP-based per capita health expenditure measured in international dollars varies from a minimum of $26.16 (in the Central African Republic) to a maximum of $949.58 (in South Africa), with a mean, median, and standard deviation of $183.78, $120.91, and $218.01, respectively. The wide distribution of most of these variables suggests that health systems in the SSA region are quite heterogeneous. We observed that, among the studied countries, South Africa, Namibia, and Eswatini had the higher values for total health expenditure per capita. On the other hand, countries such as the Democratic Republic of Congo, Central African Republic, Mozambique, and Ethiopia recorded the lowest values of total health expenditure per capita. The number of physicians per 1000 per population varies from a minimum of 0.019 in DR Congo to a maximum of 0.758 in South Africa with a standard deviation of 0.15. However, the number of nurses and midwives per 1000 people is lowest in Niger (0.137) and highest in South Africa (6.247), with a mean of 0.995. Hospital beds, used as a proxy for capital input in this study, recorded an average of 1.227 per 1000 population.

**Table 1:** Descriptive statistics of input, output, and environmental variables

Variables	Mean	Std. Dev	Minimum	Maximum
*Input variables*				
Per capita health spending (PPP)	183.76	218.01	26.16	949.58
Physicians density	0.136	0.150	0.019	0.756
Nurses and midwives’ density	0.995	1.150	0.137	6.247
Hospital beds density	1.227	1.193	0.100	6.300
*Output variable*				
UHC Index	66.78	8.59	52.38	81.21
*Environmental variables*				
GDP per capita, PPP	3253.94	2812.88	731.01	12,251.30
Educational level	0.424	0.107	0.198	0.695
Governance quality	-0.655	0.497	-1.600	0.325
Out-of-pocket spending	35.54	19.71	7.87	73.73
Government health spending	6.68	3.47	2.55	16.92
External health spending	25.82	17.94	2.41	72.41
Compulsory financing arrangement	43.77	14.75	18.94	73.40

For the environmental variables, the average literacy rate and GDP per capita are 42.4% and 3,254 international dollar-based PPP, respectively. Among the countries analyzed, out-of-pocket payment was a minimum of 7.87% of total health expenditure in Mozambique and a maximum of 73.73% in Nigeria. Domestic government health expenditure as a proportion of the total general government expenditure ranged from a minimum of 2.55% (Guinea) to a maximum of 16.93% (Kingdom of Eswatini). On average, the domestic government health expenditure as a share of total general government expenditure was. Among the studied countries, Mozambique, Malawi, and Rwanda recorded the highest levels of external health expenditure over 50% of total health expenditure. This shows a greater reliance on external healthcare financing in many SSA countries.

### Efficiency scores and health systems rankings

The efficiency scores for the selected SSA health systems in this study were estimated using the Shephard output-oriented VRS DEA model. Table 2 presents the results of the original DEA efficiency scores, bias, and bias-corrected efficiency scores for each country’s health system. We estimated the bias and bias-corrected efficiency scores using bootstrap procedures with 2000 iterations. The average non-corrected efficiency score was 0.872 (95% CI: 0.830—0.913) while the average bias-corrected efficiency score was 0.810 (95% CI: 0.770 – 0.850), with biases of 0.061 (95% CI: 0.024 – 0.099). The results show that considering the bias-corrected efficiency scores generated by the sample used for this study, health systems in SSA could potentially enhance UHC levels by at least 19% by adopting best practices with existing resources.

**Table 2:** Efficiency scores (original and bias-corrected), bias, and rankings of SSA countries

DMU	Efficiency Score (Original DEA)	Ranking	Bias	Efficiency Score (Bias-Corrected)	Ranking
Namibia	0.979	6	0.01	0.969	1
Zambia	0.977	8	0.013	0.964	2
Malawi	1	3	0.043	0.957	3
Mozambique	0.978	7	0.039	0.94	4
South Africa	0.943	10	0.009	0.934	5
Eswatini	0.937	12	0.011	0.926	6
Kenya	0.938	11	0.013	0.924	7
Rwanda	0.937	13	0.018	0.919	8
Lesotho	0.913	14	0.019	0.894	9
Uganda	0.905	15	0.014	0.891	10
Ghana	0.882	17	0.013	0.869	11
Tanzania	0.89	16	0.028	0.862	12
Burundi	1	3	0.163	0.837	13
Gambia, The	0.86	18	0.026	0.834	14
Senegal	0.843	19	0.031	0.812	15
Benin	0.831	20	0.029	0.802	16
Burkina Faso	0.827	22	0.031	0.796	17
Guinea	0.831	21	0.038	0.793	18
Congo, Rep	0.779	23	0.018	0.761	19
Cote d'Ivoire	0.755	24	0.01	0.744	20
Congo, Dem. Rep	1	3	0.262	0.738	21
Ethiopia	0.949	9	0.227	0.722	22
Mauritania	0.731	25	0.014	0.717	23
Cameroon	0.712	26	0.013	0.699	24
Nigeria	0.708	28	0.009	0.699	25
Mali	0.712	27	0.025	0.687	26
Central African Rep	1	3	0.323	0.677	27
Angola	0.675	29	0.009	0.666	28
Sudan	0.655	30	0.008	0.647	29
Niger	1	3	0.377	0.623	30
**Mean (95% CI)**	**0.872 (0.830 – 0.913)**		**0.061**	**0.810 (0.770 – 0.850)**	

The results, as presented in Table 2, show that five countries (Malawi, Burundi, Central African Republic, Niger, and DR Congo) had a non-corrected efficiency score equal to 1, which means they were operating on the efficiency frontier. However, once the biases generated by the sample used in this study were corrected, eight health systems showed a high technical efficiency score above 0.90 Malawi, Namibia, Zambia, South Africa, Rwanda, Kenya, Eswatini, and Mozambique. The last six performing health systems included Niger, Angola, Mali, Central African Republic, Nigeria, and Sudan.

### Second-stage results: Kruskal–Wallis test, bootstrap and tobit regression

**Fig. 5:**
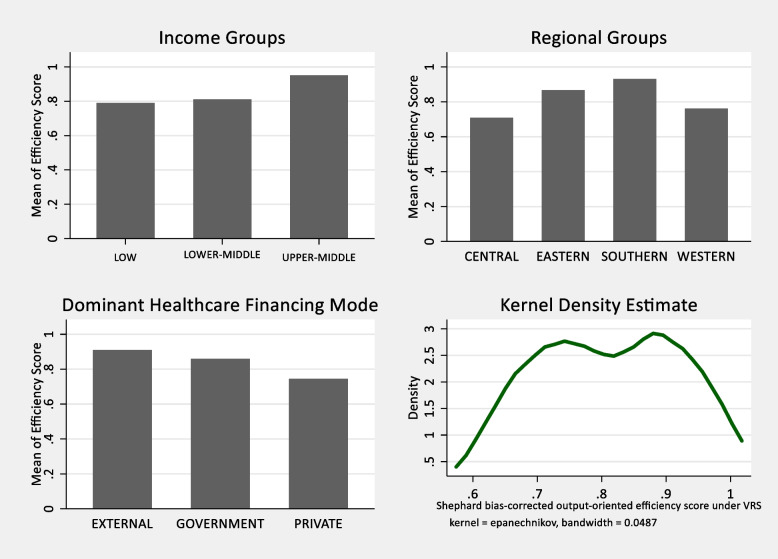
Bar graphs of efficiency scores across different categories and kernel density estimate

Before the regression analysis, we conducted the Kruskal–Wallis test to verify if there exist statistically significant differences in efficiency scores among the three different identifiable groupings within the selected health systems: income categories, regional categories, and dominant healthcare financing mode categories. We employed the Kruskal–Wallis test as a non-parametric test alternative to the parametric one-way ANOVA test since DEA is a representative non-parametric methodology. Regarding the income category variable, low-, lower-middle-, and upper-middle-income countries had mean efficiency scores of 0.790, 0.810, and 0.951, respectively (see Fig. 5). As shown in Table 3, the differences in efficiency scores among the three income groups within the sample were statistically significant at only 10 percent. This finding coincides with Ranabhat et al. [69] and McKee et al. [70] which provide evidence that all countries, irrespective of the level of economic development, can make progress towards the attainment of the UHC goal.

**Table 3:** Kruskal–Wallis test results on income, regional, and dominant financing mode

Categorical Variable	${\chi }^{2}$ Test Statistic	*P*-Value
Income groups	4.608	0.0998^*^
Regional groups	14.803	0.0020^***^
Dominant healthcare financing mode groups	13.109	0.0014^***^

^*^ < 0.10

^**^ < 0.05

^***^ < 0.01

Concerning the regional performance, the averages of bias-corrected technical efficiency scores of Central, Eastern, Southern, and Western African countries were estimated at 0.708, 0.866, 0.931, and 0.761, respectively (see Fig. 5). The differences in the performance, as shown in Table 3, were statistically significant. These results indicate that improvements can be made by countries in all the regions of sub-Saharan Africa, but the greatest efforts should be focused on countries in Central and Western Africa.

The Kruskal–Wallis test results indicate that differences in efficiency scores that exist among the three classes of health systems based on the dominant healthcare-financing mode are statistically significant (Table 3). From the results, health systems with external funding as the dominant mode for financing healthcare are the most efficient in progressing towards the UHC goal attainment. The estimated mean technical efficiency score of donor-funding-dominated health systems is 0.910 (see Fig. 5). Furthermore, systems that finance their healthcare mainly through government or public funding are more efficient than those financed largely through private funding. The estimated mean efficiency scores for public funding dominated and private funding dominated health systems are 0.860 and 0.745, respectively.

These findings sit in consonance with the relevant literature on healthcare financing in SSA. For instance, donor funding and assistance constitute a significant financing mechanism in the region and a large proportion of the external healthcare funds are invested in health insurance, user fee exemptions, and results-based financing policies that aim at achieving the UHC goal [4, 11, 71, 72]. Again, the results show that health systems that are predominantly financed through private funding are the least efficient was to be expected. The high levels of OOP health payments encapsulated in private healthcare funding potentially deny many people access to essential healthcare services and may cause catastrophic and impoverishing health payments for those who access those services [73]. Evidence shows that catastrophic health expenditure and impoverishing health expenditure remain low in countries where out-of-pocket (OOP) health expenditure is less than 15 – 20 percent of total health expenditure [74]. Many countries have implemented UHC-inspired policies which aim at reducing the high levels of out-of-pocket (OOP) health expenditure in the SSA (see Appendix C).

Smoothed bootstrap and Tobit regressions were used to relate the Shephard bias-corrected output-oriented VRS efficiency scores to the environmental variables that influence the efficiency of health systems, at least in the short to medium run, and four healthcare financing policy variables (Table 4). Since the efficiency scores were regressed on the explanatory variables in both the smoothed bootstrap and Tobit models, positive associations with the explanatory variables denote positive relation with health system efficiency. This study is one of the first to highlight the role of healthcare financing policy variables in making progress towards the attainment of SDG 3.1 in SSA.

**Table 4:** Bootstrap DEA regression results ^a^

	Model 1	Model 2	Model 3	Model 4
*Educational level*	0.538^***^	0.669^***^	0.539^***^	0.727^***^
	(0.138)	(0.133)	(0.154)	(0.148)
*Log (GDP per capita)*	-0.0836^***^	-0.129^***^	0.0218	-0.0992^***^
	(0.0176)	(0.0187)	(0.0289)	(0.0194)
*Governance quality*	0.0786^***^	0.106^***^	0.135^***^	0.121^***^
	(0.0265)	(0.0264)	(0.0273)	(0.0283)
*Out-of-pocket spending*	-0.00292^***^			
	(0.000725)			
*Domestic health spending*		0.0151^***^		
		(0.00385)		
*External health funding*			0.00425^***^	
			(0.00111)	
*Compulsory financing arr*				0.00193^**^
				(0.000882)
*Constant*	0.386^***^	0.501^***^	-0.632^***^	0.257^*^
	(0.119)	(0.126)	(0.221)	(0.140)
*Sigma *^*b*^	0.0534^***^	0.0557^***^	0.0599^***^	0.0612^***^
	(0.00749)	(0.00801)	(0.00825)	(0.00864)

^**a**^Dependent variable is the Shephard bias-corrected efficiency score

^**b**^Sigma is estimated standard deviation of the error term. Standard errors in parentheses. The coefficients are computed by 2000 bootstrap iterations

^***^ *p* < 0.01

^**^ *p* < 0.05

^*^ *p* < 0.1

Table 4 reports the results from the smoothed bootstrap (see Appendix D for the Tobit regression results). We found a strong relationship between health system efficiency and some of the environmental variables: education level, level of economic development proxied by GDP per capita, and quality of governance. The estimated coefficients of educational level and governance quality are statistically significant and positively associated with health system efficiency, implying that improvements in educational level and governance quality increase health system efficiency. These findings are consistent with previous studies on health system efficiency [31, 35, 56, 75].

On the other hand, health system efficiency is negatively associated with GDP per capita. This result must be interpreted with caution for three main reasons. First, this result stands in sharp contrast with some of the results of other studies [31, 35, 56]. Second, the selected health systems in this study are all making progress towards the UHC goal irrespective of the level of economic development, with some low- and lower-middle-income countries making greater strides as compared to the upper-middle-income countries in the sample. For instance, Malawi, Rwanda, and Mozambique (low-income countries) have efficiency scores higher than many lower-middle- and upper-middle-income countries (for example Angola, Nigeria, and South Africa). Third, the DEA methodology used here in this study suffers from some limitations, importantly it is inherently designed to measure association but not causation [38].

Regarding the relationship between healthcare financing policy variables and efficiency, the negative effects of out-of-pocket payment (OOP) on efficiency in this study coincide with Kimani et al. [73] and Kanmiki et al. [76], where a reduction in OOP was found to lower catastrophic and impoverishing healthcare expenditure (not to improve efficiency in particular). Therefore, reducing the share of OOP in the healthcare financing system structure is a sustainable major pathway for achieving the UHC goal in SSA.

The positive effect of domestic government health expenditure on efficiency favors the proposition by McIntyre et al. [77] and the Abuja Declaration (OAU, 2001) that call for an increase in government funding for healthcare to accelerate progress towards the UHC goal. The results add to the scant evidence advocating for higher domestic government health expenditure share in the general government expenditure to enhance the chances of meeting the UHC goal by the year 2030 (WHO-Africa, 2013).

The strong positive association between external funding for health and efficiency is a pointer to the significant role it plays in the SSA region. This result portends well for the attainment of the UHC goal in the SSA region in the context of the substantial inflow of donor funding for health, albeit raising the critical question about sustainability due to the volatility of this type of financing mechanism. Thus, there is a need on the part of countries in the SSA to develop innovative financing mechanisms that are sustainable in achieving and maintaining the UHC goal.

### Sensitivity analysis

**Fig. 6:**
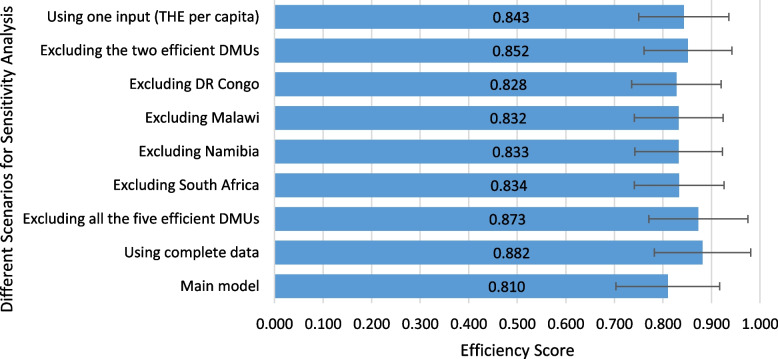
Error bars for sensitivity analysis of efficiency scores

We conducted sensitivity analyses to assess the robustness of the empirical results of this study using different combinations of input and output variables, excluding outliers and efficient DMUs. In all of these different scenarios, the average bias-corrected efficiency score ranged from 0.810 to 0.882. We found the most sensitive scenario while using the complete data on input and output variables (see Fig. 6).

Table 4 presents the estimations from the Simar and Wilson [67] bootstrap procedure based on algorithm 2. The estimated coefficients and their significance levels from the two estimation procedures produce highly similar results, conferring robustness to the empirical evidence from this study.

## Conclusion and policy implications

This paper makes a significant contribution to the literature by estimating the UHC indices of 30 SSA countries using ten tracer health service coverage and two financial risk protection indicators. The study found a cluster of countries with high UHC scores between 75 and 80: Malawi, Namibia, South Africa, Rwanda, Zambia, and Eswatini. At the other end of the spectrum, the study found a cluster of countries that scored between 50 and 58: Central African Republic, Mali, Nigeria, Cameroon, and Sudan.

The paper further examined the efficiency with which countries in the SSA are progressing towards attaining the UHC goal by the year 2030. We found that low-, lower-middle-, and upper-middle-income countries could improve their UHC scores by 29.2 percent, 13.4 percent, and 6.9 percent, respectively, without increasing the health resources at their disposal. Given that all countries in the SSA have committed to achieving the UHC goal, it is imperative that health policies should aim at health financing reforms, service coverage reforms, and more importantly improving efficiency.

This paper makes another significant contribution to the literature by examining the environmental factors and health financing policy variables that influence the efficiency with which countries in the region are progressing towards achieving the UHC goal. Concerning the environmental factors, the study found that educational level and quality of governance have a positive statistically significant association with health system efficiency. While out-of-pocket payment was found to have a negative impact, government health spending, external funding for health, and prepayment arrangement pooled for healthcare have a positive effect on health systems’ efficiency in making progress toward UHC.

## Supplementary Information

**Additional file 1.**

## Data Availability

The dataset used in this study was extracted from Health Equity and Financial Protection Indicators (HEFPI) Database, Global Health Expenditure Database (GHED), World Development Indicators (WDI), and Worldwide Governance Indicators (WGI). All are publicly available.

## Abbreviation

MDGs: Millennium Development Goals
SDGs: Sustainable Development Goals
UHC: Universal Health Coverage
SC: Health Service Coverage
SC_P_: Prevention Service Coverage Index
SC_T_: Treatment Service Coverage Index
FP: Financial Risk Protection
DEA: Data Envelopment Analysis
DMUs: Decision-Making Units
VRS: Variable Return to Scale
