# Supplementary material for: Assessing the efficiency of health systems in achieving the universal health coverage goal: evidence from Sub-Saharan Africa
Source: Health Econ Rev. 2023 May 2;13:25. doi: 10.1186/s13561-023-00433-y (PMC10152035; doi:10.1186/s13561-023-00433-y)
Supplement: Supplementary file 1 — Additional file 1. [file 13561_2023_433_MOESM1_ESM.docx]

**Appendix A:** Definitions of Health Service and Financial Protection Indicators

| **No.** | **Tracer Indicator** | **Abbr.** | **Numerator**  **(Definition of use)** | **Denominator**  **(Definition of need)** |
| --- | --- | --- | --- | --- |
| *Health Service Coverage: Prevention Indicators* | | | | |
| 1. | Family Planning | FP | Number of women age 15-49 years who are married or live in union who do not want to become pregnant and currently using any modern family planning method. | Number of women age 15-49 years who are married or live in union who desire to either prevent or delay pregnancy. |
| 2. | Four or more antenatal care visits | ANC | Number of women age 18-49 years whom for their most recent births made at least four antenatal visits in the last two years. | Number of women ages 18-49 years who gave birth in the last two years in the sample. |
| 3. | Full Immunization | IMM | Number of children age 15-23 months who receive Bacillus Calmette-Guerin (BCG), measles, 3 doses of polio, and 3 doses of Diphtheria-Pertussis-Tetanus (DPT) vaccinations. | Number of children age 15-23 months in the sample. |
| 4. | Insecticide Treated Bed-nets | ITN | Number of children under 5 years who sleep under an insecticide treated bed net. | Number of children under 5 years in the sample. |
| 5. | Water and Sanitation | WASH | Number of households with access to at least basic sanitation. | Number of households in the sample. |
| *Health Service Coverage: Treatment Indicators* | | | | |
| 6. | Treatment of Diarrhea | DIA | Number of children under 5 years with diarrhea in the 2 weeks before the survey who were given oral rehydration salts (ORS). | Number of children under 5 years with diarrhea in the 2 weeks before the survey. |
| 7. | Effective Treatment of Tuberculosis | TB | Number of TB patients who successfully completed treatment without bacteriological evidence of treatment failure. | Number of TB patients. |
| 8. | Skilled Birth Attendance | SBA | Number of women age 18-49 years whose most recent births in last 2 years were attended by any skilled health personnel. | Number of women age 18-49 years who gave birth in the last two years. |
| 9. | Antiretroviral Treatment for HIV | ART | Number of people living with HIV receiving antiretroviral treatment. | Number of people living with HIV. |
| 10. | Acute Respiratory Infection Treatment | ARI | Number of children under 5 years with cough and rapid breathing in the two weeks preceding the survey who had a consultation with a formal health care provider. | Number of children under 5 years with cough and rapid breathing in the two weeks preceding the survey. |
| *Financial Risk Protection Indicators* | | | | |
| 11. | Inverse of Catastrophic Health Expenditure | $1-CAT$ | Number of households in the survey that *did not* incur out-of-pocket (OOP) health spending exceeding 10% of the household consumption or income. | Number of households in the survey. |
| 12. | Inverse of Impoverishing Health Expenditure | $1-IMP$ | Number of households in the survey that *were not* pushed into poverty by OOP, using a poverty line of US$1.90 (in 2011 PPP). | Number of households in the survey. |

**APPENDIX B:** Estimation of Health Service Coverage (HSC) Index, Financial Protection (FP) Index, and Universal Health Coverage (UHC) Index

| COUNTRY | FAP | ANC | IMM | ITN | CPR | SCP | DIA | TB | SBA | ARI | ART | SCT | **HSC** | CAT | IMP | **FPC** | **UHC** |
| --- | --- | --- | --- | --- | --- | --- | --- | --- | --- | --- | --- | --- | --- | --- | --- | --- | --- |
| Angola | 60.5 | 49.3 | 21.5 | 21.5 | 6.3 | 24.4 | 38.0 | 34.0 | 36.0 | 52.0 | 24.0 | 35.7 | **32.5** | 87.6 | 98.0 | **92.7** | **54.9** |
| Burundi | 65.2 | 44.3 | 84.4 | 40.5 | 15.6 | 43.4 | 34.7 | 92.0 | 81.0 | 54.4 | 49.0 | 58.6 | **54.4** | 96.7 | 99.0 | **97.9** | **72.9** |
| Benin | 68.6 | 49.5 | 49.0 | 70.0 | 11.8 | 42.4 | 23.4 | 88.0 | 70.8 | 24.3 | 48.0 | 44.3 | **43.8** | 89.1 | 98.1 | **93.5** | **64.0** |
| Burkina Faso | 72.8 | 28.4 | 80.5 | 46.6 | 10.0 | 37.8 | 17.5 | 78.0 | 66.5 | 46.8 | 46.0 | 45.5 | **43.5** | 96.9 | 98.1 | **97.5** | **65.1** |
| Central African R. | 72.0 | 31.7 | 13.4 | 36.6 | 9.6 | 25.5 | 12.7 | 78.0 | 43.1 | 22.5 | 24.0 | 29.7 | **28.6** | 93.3 | 98.9 | **96.1** | **52.4** |
| Cote d'Ivoire | 67.3 | 41.2 | 34.2 | 65.9 | 13.7 | 38.6 | 14.6 | 80.0 | 63.9 | 40.6 | 34.0 | 40.0 | **39.7** | 91.2 | 98.3 | **94.7** | **61.3** |
| Cameroon | 71.8 | 49.6 | 50.7 | 51.0 | 19.4 | 44.7 | 11.5 | 84.0 | 49.7 | 26.7 | 31.0 | 33.1 | **35.7** | 89.2 | 98.1 | **93.6** | **57.8** |
| Congo, DR | 71.6 | 42.5 | 42.0 | 55.3 | 5.1 | 32.5 | 36.9 | 89.0 | 74.3 | 32.9 | 27.0 | 46.5 | **42.5** | 95.2 | 99.1 | **97.1** | **64.3** |
| Congo, Rep. | 75.9 | 71.5 | 30.3 | 65.5 | 19.9 | 46.3 | 22.5 | 71.0 | 91.0 | 23.0 | 29.0 | 39.6 | **41.2** | 95.4 | 98.9 | **97.2** | **63.2** |
| Ethiopia | 68.6 | 25.6 | 33.4 | 26.2 | 29.9 | 34.1 | 27.9 | 84.0 | 26.2 | 24.1 | 58.0 | 38.6 | **37.4** | 95.1 | 99.1 | **97.1** | **60.3** |
| Ghana | 66.9 | 81.3 | 78.2 | 53.1 | 22.7 | 55.2 | 47.5 | 85.0 | 64.6 | 50.5 | 25.0 | 50.5 | **51.7** | 98.9 | 99.8 | **99.3** | **71.6** |
| Guinea | 80.5 | 42.4 | 18.3 | 71.9 | 7.9 | 32.3 | 29.3 | 80.0 | 47.7 | 27.5 | 30.0 | 39.2 | **37.3** | 93.0 | 97.5 | **95.3** | **59.6** |
| Gambia, The | 74.2 | 75.5 | 81.2 | 49.6 | 5.9 | 42.1 | 58.5 | 82.0 | 57.1 | 59.6 | 22.0 | 51.4 | **48.9** | 99.8 | 99.9 | **99.9** | **69.9** |
| Kenya | 67.8 | 48.7 | 69.6 | 50.0 | 48.5 | 56.1 | 45.9 | 87.0 | 65.5 | 56.7 | 59.0 | 61.5 | **60.1** | 94.6 | 98.5 | **96.5** | **76.2** |
| Lesotho | 71.0 | 69.8 | 66.2 | 6.6 | 56.9 | 41.5 | 56.9 | 74.0 | 76.1 | 63.8 | 46.0 | 62.3 | **56.3** | 95.5 | 99.6 | **97.5** | **74.1** |
| Mali | 73.8 | 30.2 | 25.8 | 76.4 | 11.6 | 34.8 | 14.8 | 77.0 | 50.5 | 25.5 | 26.0 | 32.8 | **33.3** | 96.6 | 97.5 | **97.1** | **56.8** |
| Mozambique | 78.5 | 42.8 | 61.6 | 34.9 | 6.3 | 34.0 | 48.6 | 88.0 | 44.4 | 52.8 | 37.0 | 51.7 | **46.6** | 98.4 | 99.4 | **98.9** | **67.9** |
| Mauritania | 76.7 | 58.2 | 23.9 | 34.8 | 12.2 | 34.0 | 21.1 | 71.0 | 55.3 | 27.7 | 40.0 | 39.1 | **37.8** | 88.3 | 98.7 | **93.4** | **59.4** |
| Malawi | 77.7 | 46.1 | 73.9 | 39.8 | 56.9 | 57.0 | 64.4 | 81.0 | 89.4 | 68.7 | 60.0 | 71.9 | **67.8** | 95.8 | 98.7 | **97.2** | **81.2** |
| Namibia | 68.4 | 76.6 | 71.0 | 6.6 | 51.4 | 41.7 | 70.8 | 83.0 | 84.7 | 57.0 | 75.0 | 73.4 | **63.7** | 98.8 | 99.7 | **99.2** | **79.5** |
| Niger | 82.9 | 29.9 | 50.4 | 16.7 | 9.2 | 28.6 | 41.5 | 80.0 | 22.8 | 45.0 | 35.0 | 41.2 | **37.6** | 93.4 | 97.4 | **95.4** | **59.9** |
| Nigeria | 84.3 | 36.7 | 28.4 | 51.5 | 8.2 | 32.7 | 30.7 | 84.0 | 28.4 | 23.0 | 46.0 | 37.8 | **36.5** | 84.9 | 96.5 | **90.5** | **57.5** |
| Rwanda | 74.8 | 42.8 | 91.0 | 61.7 | 46.3 | 60.8 | 24.9 | 87.0 | 89.8 | 46.0 | 71.0 | 57.6 | **58.4** | 98.8 | 99.4 | **99.1** | **76.1** |
| Sudan | 71.3 | 42.3 | 46.3 | 41.1 | 8.3 | 34.3 | 18.7 | 72.0 | 67.3 | 39.7 | 8.0 | 31.0 | **31.8** | 81.6 | 97.0 | **89.0** | **53.2** |
| Senegal | 71.2 | 38.8 | 66.9 | 54.3 | 16.9 | 44.2 | 30.2 | 86.0 | 43.1 | 42.3 | 43.0 | 45.9 | **45.5** | 96.7 | 98.9 | **97.8** | **66.7** |
| Eswatini | 87.1 | 74.5 | 76.2 | 1.8 | 67.7 | 35.9 | 87.9 | 80.0 | 84.7 | 59.5 | 69.0 | 75.4 | **62.6** | 86.6 | 98.6 | **92.4** | **76.1** |
| Tanzania | 68.6 | 41.9 | 73.3 | 52.1 | 28.6 | 50.0 | 43.6 | 90.0 | 55.2 | 44.4 | 52.0 | 54.9 | **53.7** | 96.2 | 98.6 | **97.4** | **72.3** |
| Uganda | 63.0 | 58.4 | 55.9 | 60.0 | 30.8 | 52.0 | 46.6 | 75.0 | 72.4 | 69.1 | 54.0 | 62.4 | **59.6** | 84.7 | 96.8 | **90.6** | **73.5** |
| South Africa | 81.3 | 76.5 | 67.5 | 7.7 | 67.4 | 46.5 | 46.8 | 81.0 | 95.0 | 61.8 | 49.0 | 64.2 | **59.2** | 98.6 | 99.6 | **99.1** | **76.6** |
| Zambia | 69.2 | 51.1 | 66.0 | 41.2 | 39.5 | 52.0 | 63.1 | 85.0 | 59.3 | 65.8 | 66.0 | 67.3 | **63.1** | 99.7 | 99.9 | **99.8** | **79.4** |

**Notes:** **FAP** = Family Planning; **ANC** = Antenatal Care Visits; **IMM** = Full Immunization; **ITN** = Insecticide Treated Net; **CPR** = Contraceptive Prevalent Rate**; SCP** = Preventive Service Coverage Index; **DIA** = Diarrhea Treatment; **TB** = Tuberculosis Treatment; **SBA** = Skilled Birth Attendance; **ART** = Antiretroviral Treatment; **ARI** = Acute Respiratory Infection Treatment; **SCT** = Treatment Service Coverage Index; **HSC** = Health Service Coverage Index; **CAT** = Proportion of population who *did not* suffer from catastrophic health expenditure; **IMP** = proportion of population who *did not* suffer from impoverishment health expenditure; **FPC** = Financial Protection Coverage; **UHC** = Universal Health Coverage.

**Appendix C: Major UHC-Inspired Health Reforms in SSA countries between 1995 and 2015**

| Country | Description of UHC-Inspired Reforms | Year |
| --- | --- | --- |
| Angola | Created independent financial units in all 164 municipalities to increase financial access to health care services (Chol et al., 2018). | 2008 - 2010 |
| Eritrea | Developed fee retention policy for all local health facilities to enhance financial access to health care services (Chol et al., 2018). | 2006 |
| Congo, DR | Introduced donor-sponsored performance-based health financing (PBF) and reduced user fees (Miani, Mournier-Jack, and Borghi, 2018). |  |
| Ethiopia | Implemented a health extension program (HEP) that aimed at constructing health posts and posting community health extension officers to extend primary healthcare coverage to rural settings (Wagstaff et al., (2016).  Piloted Community-Based Health Insurance Scheme (CBHIS) in 13 districts and scaled it up to 185 districts in four regions (Fenny et al., 2021; Feleke et al., 2015). | 2003  2010 and 2015 |
| Kenya | Launched Health Sector Services Fund (HSSF) to finance health facilities and remove user fees (Maina and Ongul, 2014). | 2010 |
| Lesotho | Signed onto a Public-Private Partnership (PPP) between the government and a consortium of investors (called Tsepong) requiring the latter to provide a range of primary, secondary, and tertiary healthcare services as well as healthcare facilities for a lump sum of money (Hellowell, 2019). | 2008 |
| Rwanda | Introduced CBHIS (*Mutuelle de Sante*), upscaled it in 2003, and transformed it from voluntary CBHIS to a compulsory Social Health Insurance (SHI), the only formal insurance scheme in SSA with an enrollment rate of over 80% of the population (Kunda, 2014; Makaka et al., 2012; Fenny et al., 2021). | 2003 and 2010 |
| Ghana | Introduced National Health Insurance Scheme (NHIS) with premium payment exemptions for children and the poor (Kusi-Ampofo et al., 2015; Agyepong and Adjei, 2008). | 2003 |
| Malawi | Introduced free primary health care (PHC) services called Essential Health Package (EHP) at all government facilities and non-profit private facilities contracted by the Ministry of Health (Chirwa et al., 2013). | 2004 |
| Nigeria | Launched a voluntary contributory National Health Insurance Scheme to increase prepayment funds and improve financial access to health care.  Piloted a free MDG-related National Health Insurance Scheme to provide free maternal and child health (MCH) services in 78 local government areas in 12 states (Wagstaff et al., 2016). | 2004    2008 |
| South Africa | Passed National Health Act to harmonize the public and private sectors of the health system to ensure equitable provision of healthcare services and decentralized health system to the district level to implement primary health care throughout the country.  Launched government Green Paper committing the government to the comprehensive 15-year program to move the country towards UHC (Wagstaff et al., 2016). | 2004  2011 |
| Tanzania | Implemented the National Health Insurance Fund (NHIF) to offer compulsory coverage for all workers in the formal sector (Marwa, 2016).  Introduced Community Health Fund and *Tiba kwa Kadi* (TIKA) schemes targeting those in the informal sector (Mills, Ataguba, Akazili, et al., 2012). | 1999  1996 |
| Uganda | Abolished all user fees at first-level government health facilities (Xu et al., 2006). | 2001 |
| Zambia | Removed user fees in all district hospitals and health centers in rural and peri-urban areas to make healthcare services accessible to all, particularly the poor (Cheelo et al., 2010). | 2007 |

Source: Author compilation

**Appendix D: Tobit regression results**

|  | Model 1 | Model 2 | Model 3 | Model 4 |
| --- | --- | --- | --- | --- |
| Educational level | 0.418*** | 0.516*** | 0.395*** | 0.565*** |
|  | (0.104) | (0.0976) | (0.109) | (0.109) |
| Log (GDP per capita) | -0.0618*** | -0.0989*** | 0.0157 | -0.0742*** |
|  | (0.0134) | (0.0137) | (0.0204) | (0.0146) |
| Governance quality | 0.0566*** | 0.0776*** | 0.102*** | 0.0896*** |
|  | (0.0198) | (0.0187) | (0.0193) | (0.0209) |
| Out-of-pocket spending | -0.00231*** |  |  |  |
|  | (0.000525) |  |  |  |
| Domestic health spending |  | 0.0120*** |  |  |
|  |  | (0.00281) |  |  |
| External health funding |  |  | 0.00307*** |  |
|  |  |  | (0.000699) |  |
| Compulsory financing arr. |  |  |  | 0.00147** |
|  |  |  |  | (0.000651) |
| Constant | 1.250*** | 1.349*** | 0.507*** | 1.153*** |
|  | (0.0870) | (0.0912) | (0.155) | (0.106) |
| Sigma | 0.00166*** | 0.00179*** | 0.00195*** | 0.00224*** |
|  | (0.000429) | (0.000462) | (0.000503) | (0.000579) |

Notes: Dependent variable is the Shephard bias-corrected efficiency score. Sigma is the estimated standard deviation of the error term. Standard errors in parentheses. *** p<0.01, ** p<0.05, * p<0.1
